# Stratification of Amniotic Fluid Cells and Amniotic Fluid by Sex Opens Up New Perspectives on Fetal Health

**DOI:** 10.3390/biomedicines11102830

**Published:** 2023-10-18

**Authors:** Ilaria Campesi, Giampiero Capobianco, Antonella Cano, Valeria Lodde, Sara Cruciani, Margherita Maioli, Giovanni Sotgiu, Maria Laura Idda, Mariangela Valentina Puci, Margherita Ruoppolo, Michele Costanzo, Marianna Caterino, Francesca Cambosu, Andrea Montella, Flavia Franconi

**Affiliations:** 1 Department of Biomedical Sciences, University of Sassari, 07100 Sassari, Italy; antonellacano@gmail.com (A.C.); vlodde@uniss.it (V.L.); scruciani@uniss.it (S.C.); mmaioli@uniss.it (M.M.); montella@uniss.it (A.M.); 2 Laboratory of Sex-Gender Medicine, National Institute of Biostructures and Biosystems, 07100 Sassari, Italy; franconi.flavia@gmail.com; 3 Gynecologic and Obstetric Clinic, Department of Medicine, Surgery and Pharmacy, University of Sassari, 07100 Sassari, Italy; 4 Clinical Epidemiology and Medical Statistics Unit, Department of Medicine, Surgery and Pharmacy, University of Sassari, 07100 Sassari, Italy; gsotgiu@uniss.it (G.S.); mvpuci@uniss.it (M.V.P.); 5 Institute of Genetics and Biomedical Research, 07100 Sassari, Italy; m.laurai@yahoo.it; 6 Department of Molecular Medicine and Medical Biotechnology, School of Medicine, University of Naples Federico II, 80131 Naples, Italy; margherita.ruoppolo@unina.it (M.R.); michele.costanzo@unina.it (M.C.); marianna.caterino@unina.it (M.C.); 7 CEINGE—Biotecnologie Avanzate s.c.ar.l., 80145 Naples, Italy; 8 Genetics and Developmental Biology Unit, Azienda Ospedaliera Universitaria Sassari, 07100 Sassari, Italy; francesca.cambosu@aousassari.it

**Keywords:** amniotic fluid, amniotic fluid cells, sex differences, cell fate, gene expression, inflammatory indexes, metabolome, fetal health

## Abstract

Amniotic fluid is essential for fetus wellbeing and is used to monitor pregnancy and predict fetal outcomes. Sex affects health and medicine from the beginning of life, but knowledge of its influence on cell-depleted amniotic fluid (AF) and amniotic fluid cells (AFCs) is still neglected. We evaluated sex-related differences in AF and in AFCs to extend personalized medicine to prenatal life. AFCs and AF were obtained from healthy Caucasian pregnant women who underwent amniocentesis at the 16th–18th week of gestation for advanced maternal age. In the AF, inflammation biomarkers (TNFα, IL6, IL8, and IL4), malondialdehyde, nitrites, amino acids, and acylcarnitines were measured. Estrogen receptors and cell fate (autophagy, apoptosis, senescence) were measured in AFCs. TNFα, IL8, and IL4 were higher in female AF, whereas IL6, nitrites, and MDA were similar. Valine was higher in male AF, whereas several acylcarnitines were sexually different, suggesting a mitochondrial involvement in establishing sex differences. Female AFCs displayed higher expression of ERα protein and a higher ERα/ERβ ratio. The ratio of LC3II/I, an index of autophagy, was higher in female AFCs, while *LC3* gene was similar in both sexes. No significant sex differences were found in the expression of the lysosomal protein LAMP1, while p62 was higher in male AFCs. *LAMP1* gene was upregulated in male AFCs, while *p62* gene was upregulated in female ones. Finally, caspase 9 activity and senescence linked to telomeres were higher in female AFCs, while caspase 3 and β-galactosidase activities were similar. This study supports the idea that sex differences start very early in prenatal life and influence specific parameters, suggesting that it may be relevant to appreciate sex differences to cover knowledge gaps. This might lead to improving the diagnosis of risk prediction for pregnancy complications and achieving a more satisfactory monitoring of fetus health, even preventing future diseases in adulthood.

## 1. Introduction

Amniotic fluid is fundamental for the wellbeing of fetuses. It plays a crucial role in protection from mechanical traumas, maintenance of a sterile environment, and in the development of lungs and limbs [1,2], being also a reservoir of fluid and nutrients for the fetus [3]. Amniotic fluid reflects fetal health and pregnancy status; therefore, it is useful to monitor the pregnancy and fetal outcomes [4]. Transcriptome studies indicate that amniotic fluid is altered by various pregnancy complications, spontaneous preterm labor, obesity, race, and smoking [5,6,7,8]. Amniotic fluid contains fetal cells (i.e., keratinocytes, fibroblasts, neurons, glial cells, and epithelial, hematopoietic, trophoblastic, and mesenchymal cells) with stem-cell features (i.e., pluripotent differentiation ability) [9,10]. Notably, amniotic fluid cells (AFCs) have been used for fetal diagnosis since 1956 [1].

Recently, it emerged that both sex and gender can affect health and care since the beginning of life, as discussed in several articles and books [11,12,13]. Several sex differences can be found between male and female fetuses (e.g., gestational diabetes is fetal-sex-dependent), and sex affects growth trajectory (male fetuses are bigger by the first trimester of gestation), inflammatory response [14,15], and metabolomics [16].

However, only limited data are available on the impact of sex on cell-free amniotic fluid (AF) and AFCs. Insulin-like factor 3 is, for example, present only in male AFCs [17], while leptin is lower in the AF of male-carrying pregnancies [18]. However, it is not known whether inflammatory and anti-inflammatory indexes such as interleukin (IL)4, IL6, IL8, and tumor necrosis factor (TNF)α are sex-different; nevertheless, they are detected in amniotic fluid [19,20,21]. In adults, IL6 and TNFα levels display sex differences, being higher in men than in women [22]. Sex differences in the adult population were found also for anti-inflammatory IL4 [23]. However, these differences are not reported in amniotic fluid [19,20,21], but it is known that their production increases throughout normal pregnancy [24]. IL8 has a role in gestational diabetes and obesity in pregnancy [25].

Actually, the application of omics may give timely information on fetal metabolism, including the effect of maternal diet and exposure to exogenous molecules, which leads to a more precise diagnosis and treatment of developmental and metabolic conditions. For example, that AF metabolomic profile is altered in a sex-specific manner by gestational diabetes during the second trimester of gestation [8,26].

Sex differences have been described in numerous fetal cells such as human umbilical endothelial cells (HUVECs) and smooth muscle vascular cells obtained from the umbilical cord [27,28,29]. To the best of our knowledge, we do not know if the sex of fetuses influences AFC physiology. For example, very little attention has been paid to the expression of estrogen receptors (ERs). Some authors did not find ERs in AFCs or amniotic epithelia [30,31], probably due to methodological issues [31]. In full term pregnancy, other authors found ERβ in amniotic epithelial cells and fibroblasts, whereas ERα was only found in amniotic fibroblasts [31]. In human amnion-derived WISH cells, estrogens interact with specific intracellular receptors, releasing arachidonic acid [32]. As a matter of fact, the knowledge of sex differences in ER is important because they drive the expression of other numerous genes.

The three isoforms of nitric oxide synthases can be found in the amnios [33], and they produce nitric oxide (NO), an important cell-signaling molecule implicated in uterine quiescence during gestation, acting as an inflammatory agent at high concentrations as well as being implicated in preterm labor [34].

In cell wellness regulation, autophagy is a cellular “self-eating” mechanism that, through fine regulation, plays a fundamental role in degrading and recycling cellular components [35,36]. Interestingly, autophagy appears to be a sex-dependent process [27,28,37,38]: sex differences are described in HUVECs [28], in human umbilical artery smooth muscle cells [27], and in Wharton’s cells [37]. Another crucial process for cell fate is apoptosis [39], which also occurs in all placental cell types, playing a role in normal placental growth and development [40]. Apoptosis is more elevated in the male placenta and male HUVECs [41,42].

Finally, cellular senescence is implicated in maintaining pregnancy homeostasis [43] and fetal development [44]. Telomerase activity reduction causes cellular senescence and/or cellular death [45]. Authors showed that the male sex is linked to a shorter placental telomere length, being also associated with DNA methylation [46], while another study reported that telomere length does not seem to be influenced by sex [47]. Notably, so-called premature senescence may be induced independently of telomere length [48].

In light of all these considerations, this research aimed at analyzing the effect of fetal sex on a series of physiological parameters in AF and AFCs obtained from amniocentesis performed in the second trimester of pregnancy. In particular, we examined the metabolomics profile (amino acids and acylcarnitines) and inflammatory and oxidative stress biomarkers in AF, as they are strongly influenced by sex in several other cells and subjects (see above). Moreover, in AFCs, the expression of ERs and the fate of basal cells in terms of autophagy, apoptosis, and senescence were analyzed.

A deeper knowledge of AF and AFC behavior could help to reach precision medicine at a very early age [49,50], aiming to provide a guide for the direction of normal fetal growth and development and to highlight novel biomedical implications of AF and AFCs in assessing the health of the fetus or the future newborn.

## 2. Materials and Methods

### 2.1. Study Design and Participants

Each mother provided informed signed consent for the study and was accurately informed that part of their sample could be used for research purposes. All used samples were obtained from the supernumerary (unused) flask samples after cytogenetic analysis.

Mothers were healthy Caucasian women who underwent amniocentesis only for advanced age (in Italy, for women aged 35 or over, the category most at risk, the exam is generally recommended) [51,52]. Only samples free of genetic disorders after cytogenetic analysis, performed as described in Cambosu et al. [53], were used. The study was performed according to the Declaration of Helsinki.

### 2.2. AF (Cell-Depleted Amniotic Fluid) Preparation

AF samples were obtained from pregnant women at the 16th–18th week of gestation and processed at the Genetics and Developmental Biology Unit, AOU Sassari, the reference center for prenatal chromosomal analysis in Northern Sardinia, Italy. The AF was processed as previously reported [53] to obtain AF (cell-depleted). Pellets containing AFCs were suspended in CHANG Medium B Basal supplemented with CHANG Medium C Lyophilized Supplement (Technogenetics S.p.A; Milano, Italy) and 2 mM glutamine, and cultured in a CO_2_ incubator at 37 °C. The cells were cultured until sub-confluence, then expanded and used at P2 for experiments. All studied parameters in AF were assayed in duplicate.

### 2.3. Western Blotting Analysis

Protein concentration was quantified using the Quantum Protein BCA Assay kit (Euroclone, Pero, Italy) in 80% confluent male and female AFCs after cell lysis. Western blotting was performed on 25 μg of solubilized protein as previously described [28]; protein expression was evaluated using the following primary antibodies: actin (1:1000; Sigma-Aldrich, Milano, Italy), ERα, ERβ (1:1000; Thermo Fisher Scientific, Rodano, Italy), LC3 (1:1000; MBL, Eppendorf Italia, Milano, Italy), p62 (1:1000), and LAMP1 (1:1000); these were obtained from Cell Signaling Technology (Milano, Italy) as primary antibodies.

After 1 h incubation with horseradish peroxidase (HRP)-conjugated secondary antibody (1:2000; Cell Signaling Technology, Milano, Italy), the binding was detected by chemiluminescence with the Bio-Rad ChemiDoc instrument (Bio-Rad, Milano, Italy). Band volume analysis was performed using the Image Lab 4.0 software (Bio-Rad, Milano, Italy). A pilot study (5 samples of each sex) suggested that in female and male AFCs, the expression of α-actin was very similar (827,366.79 ± 578,040.86 optical density (OD) and 839,428.71 ± 713,143.57 OD for females and males, respectively; *p* = 0.49). Therefore, it was used for the normalization of Western blot analysis.

### 2.4. RNA Isolation, Reverse Transcription (RT)-Quantitative (q)PCR Analysis

RNA was isolated from AFCs using the TriPure isolation reagent (Roche, Merk Life Science, Milano, Italy). First-strand cDNA synthesis was performed using Maxima Reverse Transcriptase (Thermo Fisher Scientific, Rodano, Italy) and random hexamers, and was subsequently analyzed by quantitative (q)PCR using SYBR Green mix (Kapa Biosystems, Merk Life Science, Milano, Italy). The relative mRNA expression levels were calculated by the 2−ΔCt method, and *GAPDH* mRNA levels were used for normalization. Pilot experiments showed that the housekeeping gene *GAPDH* did not differ between male and female cells (17.23 ± 1.38 mean Ct for females and 17.09 ± 0.35 for males, N = 5 for each sex; *p* = 0.80). Gene-specific primer pairs are listed in Supplementary Table S1.

### 2.5. Dosage of Cytokines

TNFα, IL6, IL8, and IL4, were measured using commercial kits (DuoSet human TNFα, DuoSet human IL6, DuoSet human IL4, and DuoSet human IL8 ELISA kits, R&D Systems, Milano, Italy) according to the manufacturer’s instructions.

### 2.6. Malondialdehyde (MDA) Determination in AF

MDA was detected as previously described [54]. The quantification was performed at 535 nm using a calibration curve built with concentrations of MDA ranging from 50 to 5 μM.

### 2.7. Nitrite Determination in AF

Nitrites, the final product of NO metabolism, were measured in 50 µL of AF using the Griess reaction [55]. Nitrite concentrations were calculated on a standard curve of sodium nitrite ranging from 50 to 1 μM.

### 2.8. Caspase 3 and Caspase 9 Activities in AFC

Activities of caspase-3 and -9 were determined using caspase assay kits (Caspase-3 and Caspase-9 Colorimetric Assay Kit; BioVision, Inc., Waltham, MA, USA) on 75 μg of solubilized proteins obtained from AFCs. Staurosporine (1 µM for 3 h) was used as a positive control, and the activity was calculated as the percentage in comparison with staurosporine (100%).

### 2.9. Senescence-Associated β-Galactosidase Staining (SA-βGal)

Cell senescence was evaluated using a SA-β Galactosidase Staining Kit (Cell Signaling Technology, Euroclone, Milano, Italy). Briefly, cells were fixed for 10–15 min at room temperature and then stained with β-Galactosidase Staining Solution ON at 37 °C. The SA-βGal activity was detected by an inverted microscope (magnification 10× bright field), and the number of positively blue-stained cells was calculated as the percentage of the total number of cells using ImageJ software analysis (version 1.8.0, National Institutes of Health, Bethesda, MD, USA).

### 2.10. Catalytic Subunit of the Telomerase Reverse Transcriptase (TERT) Activity Detection

TERT was evaluated by real-time PCR using the TRAPeze^®^ Kit RT Telomerase Detection Kit (Millipore, Milano, Italy) in a CFX-96 Thermal Cycler (Bio-Rad, Segrate, Italy) according to manufacturer’s instructions. Cells were lysed, and protein concentrations were determined using a Nanodrop instrument (Thermo Fisher Scientific, Rodano, Italy). The PCR amplification conditions were 30 °C for 30 min, 95 °C for 2 min, and then 94 °C for 15 s, 59 °C for 60 s, and 45 °C for 10 s for 45 cycles, using a mix composed of a 5× TRAPeze^®^ RT reaction mix, Taq polymerase (5 units/μL), nuclease-free water, and samples, for a final volume of 20 µL. The value of each sample was normalized to the cycle threshold (Ct) of the standard curve generated from the control reaction mix included in the kit. All samples were processed in triplicate.

### 2.11. Amino Acid and Acylcarnitine Profiling by Targeted LC-MS/MS

AF samples were processed by liquid chromatography–tandem mass spectrometry (LC-MS/MS) for metabolite identification and quantification [56]. The sample preparation and the analysis were performed as published [16,57], with some adjustments. After a short centrifugation (250 rcf, 5 min, 4 °C) to remove floating cells, 10 µL of AF was spotted on a filter paper, and metabolites were extracted using 200 µL of methanol containing stable isotope-labeled amino acids and acylcarnitine standards [16]. AF and standard metabolites were derivatized using 80 µL of n-butanol/3 N HCl (30 min, 65 °C) and dried under nitrogen flow. A solution of acetonitrile/water (70:30) with 0.05% formic acid was added for injection onto the LC-MS/MS system composed of a 1260 Infinity II HPLC (Agilent Technologies, Waldbronn, Germany) and an API 4000 triple quadrupole mass spectrometer (Applied Biosystems-Sciex, Toronto, ON, Canada). Acylcarnitines were detected using the precursor ion scan mode, and the complete list of all analyzed acylcarnitines is reported in Table S2. Amino acids were identified by neutral loss scan or multiple reaction monitoring (MRM), as reported [16]. Analyte quantification was achieved using the ChemoView v1.2 software comparing analytes and standards areas.

### 2.12. Statistics

Data are displayed as the mean (standard deviation, SD) or median (interquartile range, IQR). Male and female cells were compared with the unpaired Student’s *t* or Mann–Whitney test when variables showed a normal or non-normal distribution, respectively. Qualitative variables were summarized by absolute and relative (percentage) frequencies and analyzed by Fisher exact test. A *p*-value < 0.05 was considered statistically significant. For cluster analysis, the first step was to identify the set of features of interest based on clinical expertise and principal components analysis. Hierarchical cluster analysis with Euclidean distance as a dissimilarity metric and complete linkage was implemented to define homogenous groups (i.e., clusters) using STATA 13 software. Other statistical computations were performed through Sigma-Stat 3.1 software (Systat Software, Erkrath, Germany).

## 3. Results

### 3.1. Population Characteristics

Samples were obtained from the AF of 22 healthy Caucasian female fetuses and 20 healthy Caucasian male fetuses whose mothers were well matched for age (mean ± SD: 36.9 ± 4.01 years and 36.1 ± 4.6 years for mothers of female and male fetuses, respectively; *p* = 0.87) and gestational age at the time of sampling (mean ± SD: 16.5 ± 0.9 weeks for female fetuses and 16.6 ± 0.6 weeks for male fetuses; *p* = 0.92).

### 3.2. ER Protein Expression in AFCs

**Figure 1:**
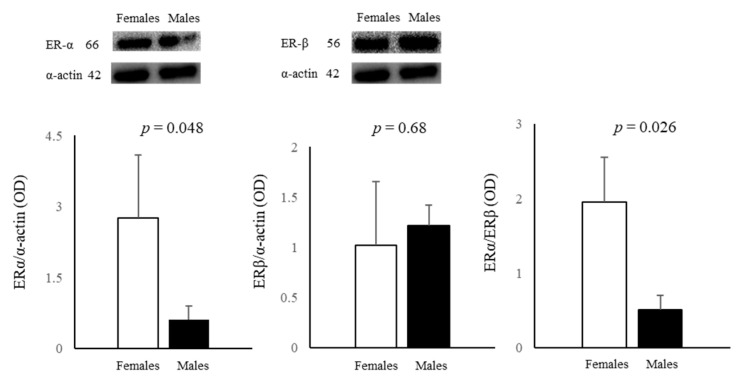
Influence of sex on the expression of ERα and ERβ proteins in female and male AFCs. Data are the mean ± SD of three samples for each sex.

Both ERα and ERβ proteins were detected in male and female AFCs. In detail, female cells had significantly higher ERα levels (4.5 fold), whereas ERβ had a similar expression in cells obtained from both sexes (Figure 1). The ERα/ERβ ratio was significantly higher in female cells (Figure 1).

### 3.3. Cell Fate: Autophagy, Apoptosis, and Senescence

**Figure 2:**
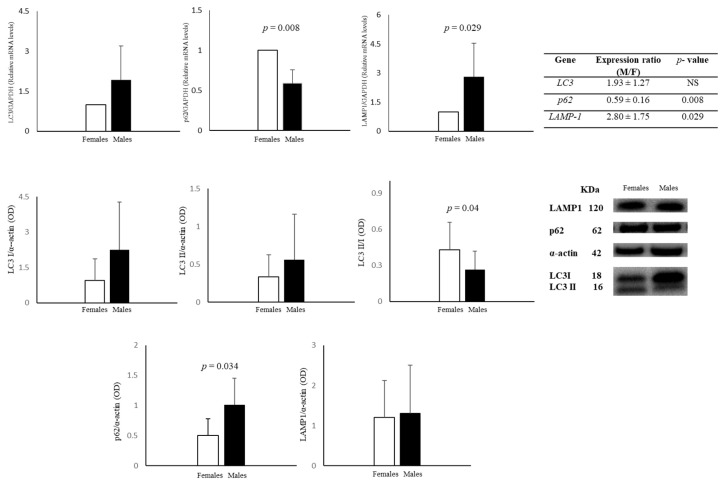
Relative gene and protein expression of LC3, p62, and LAMP1 in female and male AFCs. Data represents the mean ± SD of 5 samples for each sex, gene, and protein.

Autophagy was assayed by measuring the expression of following proteins and their gene expression: LC3I, its delipidated form LC3II, the ratio LC3II/LC3I, LAMP1, and p62 (Figure 2). Expression of *LC3* gene was similar in AFCs from both sexes, while the gene of the lysosomal protein *LAMP1* was upregulated (ratio M/F: 2.80 ± 1.75) in male cells. Finally, gene expression of *p62* was significantly downregulated (ratio M/F: 0.59 ± 0.16) in male cells (Figure 2). LC3I and LC3II protein levels did not present significant differences between male and female AFCs, although they were higher in male cells than in female ones. Interestingly, the LC3II/I ratio was significantly higher (about 62%) in female AFCs than in male ones, indicating a higher degree of constitutive autophagy in female cells. The expression of the lysosomal protein LAMP1 did not differ between male and female cells, whereas the gene was more expressed in male cells. Notably, female cells had a lower level of p62 protein and higher gene expression than male cells.

**Figure 3:**
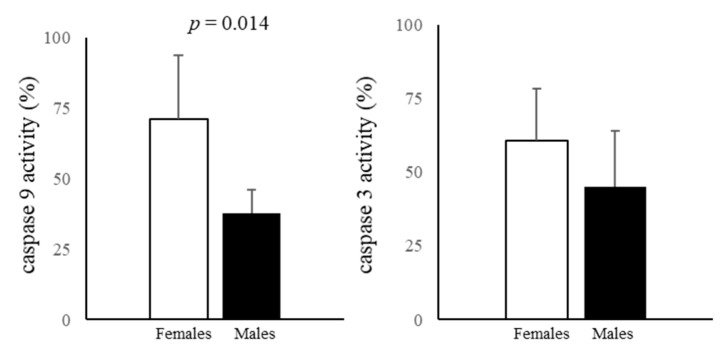
Caspase 9 and caspase 3 activities measured in male and female AFCs using DEVD-pNA and LEHD-pNA as substrates for caspase 3 and 9, respectively. Data represents the mean ± SD of five samples for each sex.

The activity of the initiator caspase 9 was significantly higher in female AFCs. The activity of the executioner caspase 3 was higher in female AFCs, whereas the sex difference was not statistically significant (Figure 3).

**Figure 4:**
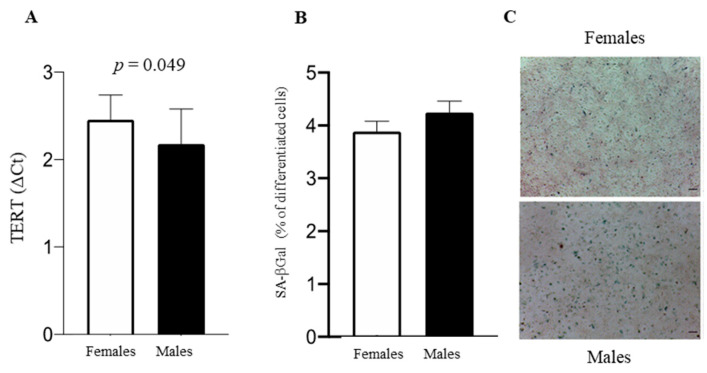
(**A**) TERT in male and female AFCs, expressed as mean ± SD of four experiments for each sex. (**B**) SA-βGal activity quantification. (**C**) Representative images of SA-βGal staining. The number of blue-positive cells was calculated using ImageJ. Data are expressed as mean ± SD. Scale bar = 100 μm.

TERT was more elevated in female cells (Figure 4A); the senescence associated with SA-βGal staining (Figure 4B,C) was similar in male and female cells.

### 3.4. Nitrite and MDA Determination in AF

**Figure 5:**
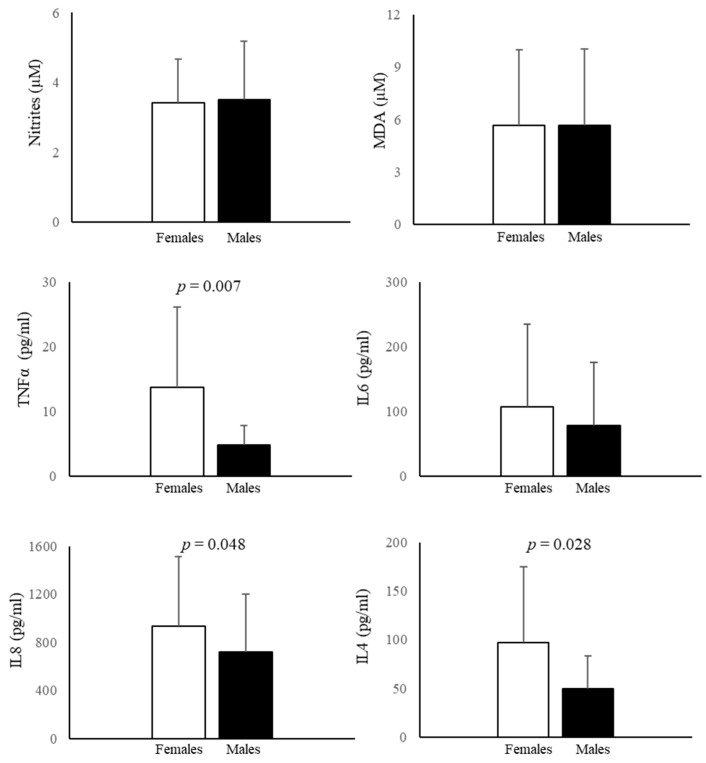
Nitrite, MDA, IL6, IL8, TNFα, and IL4 levels in AF. Data represent the mean ± SD of at least 15 samples for each sex and were analyzed in duplicate.

Nitrite levels, products of NO metabolism, and MDA, a lipid peroxidation index, were not different between female and male AF (Figure 5).

### 3.5. Cytokine Measurement in AF

Both in male and female AF, IL6, IL8, TNFα, and IL4 were detectable. were detectable. In none of the tested samples IL6 exceeded the cut-off value (≥2.6 ng/mL), which indicates intra-amniotic inflammation [58], and no significant sex differences emerged when IL6 was analyzed (Figure 5). Values of IL8 were in line with those previously measured by others [59] and showed the highest concentration followed by IL6, IL4, and TNFα. In female AF, IL8, TNFα, and IL4 were significantly higher than in male AF (Figure 5). The most evident difference was found for TNFα (185.7%), followed by IL4 (95.4%) and IL8 (30.4%).

### 3.6. Amino Acid and Carnitine Levels in AF

**Figure 6:**
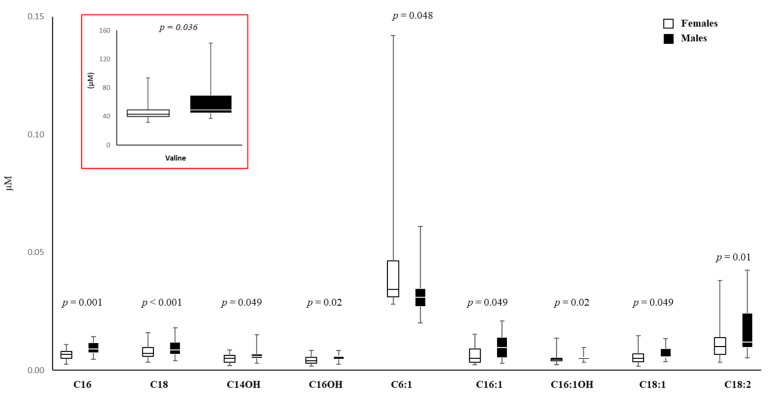
Box plots of the sex-divergent metabolites in AF from female and male cells. The horizontal line across the box represents the median, and the box comprises the first and the third quartiles. The vertical lines represent the minimum and the maximum values. Sample size for each group is reported in Table S2. The red box represents a reduction of the box plot necessary due to the different scales on the Y-axis.

A targeted metabolomics approach was used to measure the AF metabolome, profiling 50 compounds through liquid chromatography–tandem mass spectrometry (LC-MS/MS). Amino acid levels (Ala, Val, Xle, Met, Phe, Tyr, Asp, Glu, Gly, Orn, Cit, Arg) were higher in male AF by about 20% (Supplementary Table S2); but only Val was statistically significantly higher in male AF than in female AF (Figure 6).

AF levels of free carnitine (median value: 6.07, IQR: 5.22–8.27 for females, n = 22; median value: 6.16, IQR: 5.34–10.05 for males; *p* = 0.65, n = 20), total esterified carnitines (median value: 4.37, IQR: 3.69–5.02 for females; median value: 3.96, IQR: 3.71–5.09 for males; *p* = 0.84), and the ratio total esterified/free carnitine (median value: 0.70, IQR: 0.61–0.75 for females; median value: 0.65, IQR: 0.55–0.73 for males; *p* = 0.42) were similar (Supplementary Table S2).

Finally, levels of acylcarnitines C16, C18, C14OH, C16OH, C16:1, C16:1OH, C18:1, and C18:2 were higher in male AF (Figure 6), and the differences ranged from 11% to 44%. Only a medium-chain acylcarnitine C6:1 was significantly higher in female AF (+29%) (Figure 6).

### 3.7. Cluster Analysis of Metabolomic Parameters

Cluster analysis showed two main clusters (Supplementary Table S3; Supplementary Figure S1): cluster 1 comprised 57.6% females and 42.4% males, whereas cluster 2 comprised 33.3% females and 66.7% males. Subjects in cluster 2 had higher amino acid concentrations, except for Tyr and Asp, and higher levels of free carnitine, total esterified carnitines, and several acylcarnitines (C2, C3, C4, C5, C6, C5OH, C6DC, C6:1, and C10:2) than those in cluster 1.

## 4. Discussion

This investigation systematically studied the influence of sex during prenatal life (16th–18th week of gestation) in AF and AFCs, studying 56 and 12 parameters, respectively. Globally, our data evidenced that: (i) sex plays a crucial role in physiology during prenatal life, influencing the studied parameters both in AF and AFCs; (ii) the main sex differences observed in AF are linked to the immune system and acylcarnitine levels, indicating that inflammation and mitochondrial activity are more susceptible to sex effects, while lipid peroxidation and NO are not influenced; (iii) in AFCs, sex differences affect the gene expression of *p62* and *LAMP1*, while *LC3* is not affected by sex; (iv) the expression of proteins linked to cellular fate and ERs displays numerous sex differences, indicating that their expression is linked to sex. Globally, it is here confirmed that sex differences start in prenatal life and can be observed in AFCs and AF. The influence of sex on amniotic fluid is still neglected, as for many other biological fluids [60], although a very recent proteomic study shows a sex-specific regulation of protein expression in Down syndrome fetuses [61]. Significant differences in inflammation biomarkers and acylcarnitine values were found in the present study. In the AF, the largest sex effect was observed in the inflammatory profile, with pro-inflammatory TNFα (185.7%) and IL8 (30.4%) levels higher in female than in male AF, while in line with Poggi and collaborators [19], IL6 does not diverge. According to our results, anti-inflammatory IL4, which has beneficial effects on features related to type II immunity [62], was lower (95.4%) in male cells. Therefore, our findings are not in line with previous results obtained in the amniotic fluid of at-term pregnancies, where IL6 and TNFα levels were higher in male amniotic fluid [63], suggesting the importance of gestational age, as already described [20,21]. Thus, our results suggest that the amniotic fluid in pregnancies carrying female fetuses exhibit greater pro-inflammatory cytokine levels, which are attenuated by an increase in anti-inflammatory IL4 levels, than the amniotic fluid of male fetuses. Comprehensive metabolomic profile analysis would lead to new insights into sex differences in the pathogenesis of inflammatory and mitochondrial diseases. These results could suggest sex differences in clinical manifestations and could help in reaching personalized diagnosis and treatment of mitochondrial and inflammatory diseases. Furthermore, if these differences are confirmed by larger studies, they also may suggest that reference values could be sex-divergent, at least for some cytokines and some acylcarnitines.

The AF target metabolome shows that, among tested amino acids, only Val is statistically significantly higher in male AF, by about 25%, and this could be of some importance for the diagnosis of non-immune hydrops, which is based on Ala, Gly, and Val [64].

Acylcarnitine production mainly occurs in the mitochondria and plays a crucial role in β-oxidation and other mitochondrial functions [65]; although they are used for the prenatal diagnosis of metabolic diseases to prevent multi-organ damage, it is not currently known if their levels in AF are influenced by sex [66]. In the present study, C16, C18, C14OH, C16OH, C16:1, C16-OH, C18:1, and C18:2 values were significantly higher in male AF. Indeed, the medium-chain acylcarnitine C6:1, which is also metabolized in peroxisomes [67], was significantly higher in female AF. The sex differences in the levels of acylcarnitines suggest differential mitochondrial transport of fatty acids in female and male fetuses, indicating that in very early life, mitochondrial physiology is sex-different, as in postnatal life [65,68]. In conclusion, fetal sex may influence the AF metabolome, especially when acylcarnitines are taken into consideration, and this might help in the diagnosis and therapy of mitochondrial diseases.

Cluster analysis of metabolomic data revealed two main clusters: in cluster 1, female AF (57.6%) prevailed over males (42.4%), while males prevailed over females in cluster 2, confirming the importance of sex.

Male and female AFCs express both ERα and ERβ proteins, with ERα more highly expressed in female cells (4.5-fold), while ERβ expression is similar in the two sexes. ERβ and ERα are detected in other prenatal tissues and cells, such as at-term placenta, amniotic epithelial cells, fibroblasts, extra-villous trophoblasts [69], and HUVECs [28]. Both the higher ERα expression, which is implicated in autophagy in an mTOR-independent manner [70], and the upregulation in female AFCs of the multifunctional adaptor protein p62, which causes p62 degradation when interacting with LC3 [35], might explain why autophagy is higher in female AFCs than in male AFCs. LAMP1 protein expression did not sexually diverge in AFCs, suggesting no significant sex differences in lysosomes. These data confirm that also in AFCs, the autophagic process is highly sex-sensitive, similarly to that in other cells and tissues [28,37,38].

Both TERT and SA-βGal activities have a role in cellular senescence, impacting different mechanisms [71,72]. Female AFCs are less senescent only when TERT is considered because SA-βGal does not vary between sexes. SA-βGal is linked to the lysosomal compartment [73], and the lysosomal protein LAMP1 does not diverge between sexes. Thus, the effect of sex on senescence seems to be limited to replicative senescence, which occurs as a result of telomere truncation [74] being absent when the senescence marker is SA-βGal activity.

*LC3* gene expression is similar in both sexes, whereas *p62* gene is more expressed in female cells and *LAMP1* gene is more expressed in male cells. Specifically, *p62* and *LAMP1* genes seem to be less or more efficient regarding relative protein expression in female AFCs than in male AFCs. The complex journey from gene to protein may often lead to a discrepancy between the expression of cognate protein and gene. This journey, which is pivotal for physiological responses [75], appears to be influenced by sex, at least for p62 and LAMP1. The influence of sex on LAMP1 was already seen in the brain [76].

Caspase 9 is higher in female cells, while caspase 3 is similar in both sexes. Caspase 3 activation occurs independently from caspase 9 or mitochondrial cytochrome c release and involves the extrinsic pathway through the death receptors [77]; the fact that it is equally active in male and female AFCs further underlines the crucial role of mitochondria in the establishment of sex differences. Caspase 9 plays a crucial part in intrinsic apoptosis through the release of cytochrome c from the mitochondria [78] and also plays a role in the regulation of autophagy induced by amino-acid starvation [79], and female AFCs are more autophagic. In female AF, amino acid levels are slightly lower (the difference is not statistically significant) except for Val. This condition could mimic minimal amino-acid starvation, which might lead to an increase in caspase 9 activity, elevating autophagic flux.

These results emphasize the importance of studying sex differences in prenatal life, as male and female AF and AFCs have distinct profiles; sex effects start early and are associated with different parameters; therefore, knowledge of the influence of sex is crucial in the evaluation of fetal health. The fetus, in fact, goes through critical periods during gestation in which it is actively shaped by the environment, so that the knowledge of sex-related differences sheds light on new medical perspectives and facilitates the path that leads to precision medicine in prenatal life. Moreover, our data suggest the need for sex-specific reference values, especially those related to inflammatory indexes and acylcarnitines (indicative of the mitochondrial status), when they are used for diagnostic purposes. The identification of sex-specific reference values in prenatal life is warranted, as these values could help to ascertain errors in the developmental trajectory [80], and levels of acylcarnitine may help to identify drug-induced mitochondrial dysfunction [80].

Future research should focus on further sex-specific parameters and molecular mechanisms, extending the analyses to all stages of pregnancy to better address and inform clinical practice. Finally, considering that AFCs are also used in regenerative medicine [81], the knowledge of sex differences could also have a therapeutic value.

## 5. Conclusions

The present study supports the idea that sex differences start very early in life, influencing specific parameters. Although currently, offspring sex is not considered in most prenatal diagnostic strategies, our results suggest that it may be important to consider sex differences to cover knowledge gaps, which might lead to improvement in the diagnosis of risk prediction to avoid pregnancy complications and to improve fetal health monitoring, even preventing future diseases in adulthood.

## 6. Strengths and Limitations

Although amniocentesis is associated with a risk of miscarriage (the risk of fetal loss is about 1 in 1000) [82], this procedure is still considered by some authors the gold standard for diagnosing fetal chromosomal abnormalities, being more accurate than non-invasive prenatal DNA testing [83].

Moreover, using a metabolomic approach, it was reported that amniotic fluid is very useful for the prenatal diagnosis of some metabolic diseases such as methylmalonic acidaemia and ornithine transcarbamylase deficiency [84]. In view of the improvements offered by metabolomic analysis, our basic research study helps to understand whether fetus sex affects biomarker levels in order to improve diagnosis in both sexes.

Here, amniocentesis was performed only once during gestation, namely in the second trimester. Therefore, the possible variations that may occur during pregnancy are not identified. Multiple amniocentesis performed in different periods of pregnancy could provide a more comprehensive picture of the behavior of AF and AFCs. As already mentioned, amniocentesis is associated with a risk of miscarriage; therefore, repeated sampling can present some safety problems. Despite this, our findings are innovative because sex differences in AF and AFCs were evaluated in a relatively early stage of pregnancy when amniocentesis is performed to evaluate genetic damage. Future research will therefore also focus on the relationships between maternal and fetal status and provide a more complete view.

## Data Availability

Data supporting the findings of this study are available from the corresponding author upon reasonable request.

## Supplementary Materials

The following supporting information can be downloaded at: https://www.mdpi.com/article/10.3390/biomedicines11102830/s1, Figure S1: Dendrogram for hierarchical cluster analysis of metabolomic data; Table S1: Gene-specific primer pairs; Table S2: Metabolomics analysis in AF; Table S3: Cluster analysis on metabolomics.
