# Supplementary material for: Stratification of Amniotic Fluid Cells and Amniotic Fluid by Sex Opens Up New Perspectives on Fetal Health
Source: Biomedicines. 2023 Oct 18;11(10):2830. doi: 10.3390/biomedicines11102830 (PMC10604128; doi:10.3390/biomedicines11102830)

Supplementary material

Supplementary Table 1. Gene-specific primer pairs

RT-(q)PCR primers	Primer Sequences (5′ - 3′)
SQSTM1 (p62)	FW: GGGGACTTGGTTGCCTTTT
	RV: CAGCCATCGCAGATCACATT
LC3	FW: GAGAAGCAGCTTCCTGTTCTGG
	RV: GTGTCCGTTCACCAACAGGAAG
LAMP1	FW: CTCTAATGTCTGCAGCTCAAGG
	RV: TGTACACAGCGCAGAACAGG
GAPDH	FW: TGCACCACCAACTGCTTAGC
	RV: GGCATGGACTGTGGTCATGAG

Supplementary Table 2. Metabolomics analysis in AF

	Females (N=22)	Males (N=20)	P value
**Ala**	76.86 (67.24-89.90)	82.19 (71.40 105.11)	0.19
**Val**	42.98 (39.41-49.66)	49.22 (44.53-70.45)	**0.036**
**Xle**	26.77 (23.68-31.26)	30.42 (26.03-40.51)	0.10
**Met**	5.98 (5.56-7.05)	6.42 (5.74-8.51)	0.49
**Phe**	19.84 (17.85-22.09)	20.2 (17.47-23.41)	0.95
**Tyr**	18.08 (16.65-21.49)	19.30 (16.25-22.76)	0.97
**Asp**	5.27 (4.88 -6.36)	4.90 (4.57-5.76)	0.30
**Glu**	82.92 (75.66-100.44)	77.73 (69.87-102.29)	0.49
**Gly**	62.59 (50.21-69.80)	58.21 (43.18-79.95)	0.83
**Orn**	6.36 (6.05-8.35)	6.86 (5.74-10.42)	0.80
**Cit**	3.48 (3.11 4.00)	3.29 (2.82-4.42)	0.71
**Arg**	10.45 (8.07-12.01)	10.35 (8.58-14.76)	0.57
**C0**	6.07 (5.22-8.27)	6.16 (5.34-10.05)	0.65
**C2**	3.60 (2.98-4.14)	3.21 (2.93-4.14)	0.65
**C3**	0.19 (0.15-0.22)	0.16 (0.14- 0.19)	0.19
**C4**	0.13 (0.11-0.17)	0.15 (0.11-0.22)	0.46
**C5**	0.012 (0.009-0.014)	0.012 (0.012-0.014)	0.16
**C6**	0.064 (0.056- 0.076)	0.077 (0.058-0.088)	0.90
**C8**	0.026 (0.02- 0.029)	0.025 (0.02-0.029)	0.63
**C10**	0.014 (0.013- 0.017)	0.016 (0.015-0.017)	0.90
**C12**	0.025 (0.018-0.035)	0.022 (0.018-0.028)	0.14
**C14**	0.012 (0.011 0.015)	0.013 (0.012-0.016)	0.16

**C16**	0.015 (0.011-0.022)	0.016 (0.012-0.018)	**0.001**
**C18**	0.015 (0.012 0.021)	0.015 (0.012-0.019)	**<0.001**
**C3DC**	0.017 (0.014 0.02)	0.018 (0.015-0.02)	0.22
**C4DC**	0.015 (0.013-0.019)	0.015 (0.013-0.017)	0.07
**C5DC**	0.006 (0.004-0.007)	0.006 (0.005-0.007)	0.58
**C6DC**	0.008 (0.007-0.010)	0.009 (0.008-0.012)	0.80
**C8DC**	0.013 (0.009-0.02)	0.013 (0.010-0.015)	0.94
**C10DC**	0.006 (0.005-0.009)	0.007 (0.006-0.01)	0.64
**C4OH**	0.005 (0.004-0.007)	0.005 (0.004-0.007)	0.46
**C5OH**	0.007 (0.006-0.008)	0.008 (0.006-0.01)	0.09
**C6OH**	0.007 (0.005-0.009)	0.008 (0.006-0.009)	0.60
**C12OH**	0.005 (0.003-0.009)	0.01 (0.005-0.014)	0.30
**C14OH**	0.007 (0.005-0.008)	0.009 (0.007-0.012)	**0.049**
**C16OH**	0.021 (0.02-0.023)	0.021 (0.019-0.0234)	**0.02**
**C18OH**	0.004 (0.003-0.006)	0.006 (0.005-0.006)	**0.07**
**C5:1**	0.005 (0.004-0.007)	0.006 (0.005-0.009)	0.58
**C6:1**	0.007 (0.006-0.01)	0.009 (0.007-0.01)	**0.048**
**C8:1**	0.0045 (0.004-0.0057)	0.005 (0.004-0.006)	0.36
**C10:1**	0.0165 (0.015-0.02)	0.015 (0.012-0.019)	0.99
**C12:1**	0.0103 (0.009-0.012)	0.0107 (0.009-0.012)	0.38
**C14:1**	0.034 (0.03-0.047)	0.031 (0.03-0.036)	0.55
**C16:1**	0.014 (0.011-0.023)	0.014 (0.011-0.017)	**0.049**
**C16:1OH**	0.012 (0.011-0.015)	0.014 (0.012-0.016)	**0.02**
**C18:1**	0.006 (0.005-0.009)	0.008 (0.006-0.01)	**0.049**
**C18:1OH**	0.005 (0.003-0.006)	0.006 (0.005-0.007)	0.37
**C10:2**	0.005 (0.004-0.005)	0.005 (0.004-0.006)	0.14
**C14:2**	0.004 (0.003-0.005)	0.005 (0.004-0.007)	0.10
**C18:2**	0.01 (0.0067-0.014)	0.01 (0.0093-0.024)	**0.01**
**Total esterified carnitines**	4.37 (3.69-5.02)	3.96 (3.71-5.09)	0.84
**Total esterified/C0**	0.69 (0.61-0.75)	0.65 (0.55-0.73)	0.26

Data is reported as medians (IQR)

Supplementary Table S3. Cluster analysis on metabolomics

Parameters	Cluster 1 (n = 33)	Cluster 2 (n = 9)	P-value
**Females, n (%)**	19 (57.6)	3 (33.3)	0.27
**Ala**	76.9 (67.2-89.9)	107.4 (82.19-156.5)	0.003
**Val**	43.7 (40.3-49.7)	82.1 (49.1-93.7)	0.001
**Xle**	26.9 (24.2-31.3)	47.5 (31.9-59.4)	0.001
**Met**	5.8 (5.5-6.9)	8.9 (7.2-12.6)	0.004
**Phe**	18.5 (17.3-21.5)	27.6 (22.1-37.3)	0.0004
**Tyr**	27.6 (22.1-37.3)	24.89 (20.9-40.9)	0.001
**Asp**	5.0 (4.7-5.7)	5.0 (4.7-5.7)	0.002
**Glu**	77.7 (72.0-86.3)	113.3 (83.8-128.4)	0.01
**Gly**	53.9 (45.8-63.1)	84.1 (82.8-98.0)	<0.0001
**Orn**	6.4 (5.9-7.6)	11.5 (8.4-15.9)	0.001
**Cit**	3.19 (2.8-3.6)	4.9 (4.1-5.8)	0.0002
**Arg**	9.5 (8.0-11.2)	16.5 (12.2-17.8)	0.0003
**C0**	6.1 (5.2-7.4)	10 (6.2-13.0)	0.02
**C2**	3.2 (2.9-4.0)	4.9 (3.4-4.9)	0.005
**C3**	0.16 (0.13-0.19)	0.21 (0.18-0.26)	0.02
**C4**	0.13 (0.11-0.17)	0.24 (0.18-0.26)	0.0002
**C5**	0.07 (0.06-0.08)	0.09 (0.08-0.12)	0.02
**C6**	0.02 (0.02-0.03)	0.03 (0.03-0.04)	0.02
**C8**	0.02 (0.02-0.03)	0.03 (0.02-0.04)	0.08
**C10**	0.01 (0.01-0.02)	0.02 (0.02-0.02)	0.09
**C12**	0.01 (0.01-0.01)	0.01 (0.01-0.01)	0.37
**C14**	0.01 (0.01-0.01)	0.01 (0.01-0.01)	0.95
**C16**	0.01 (0.01-0.01)	0.01 (0.01-0.01)	0.70
**C18**	0.01 (0.01-0.01)	0.01 (0.01-0.01)	0.35
**C3DC**	0.01 (0.01-0.02)	0.01 (0.01-0.02)	0.28
**C4DC**	0.02 (0.01-0.02)	0.02 (0.02-0.02)	0.03
**C5DC**	0.02 (0.01-0.02)	0.02 (0.02-0.02)	0.09
**C6DC**	0.01 (0.01-0.01)	0.02 (0.01-0.02)	0.03
**C8DC**	0.01 (0.01-0.01)	0.01 (0.00-0.01)	0.27

**C10DC**	0.02 (0.02-0.02)	0.02 (0.02-0.02)	0.78
**C4OH**	0.02 (0.01-0.02)	0.02 (0.02-0.02)	0.22
**C5OH**	0.02 (0.01-0.02)	0.02 (0.02-0.03)	0.01
**C6OH**	0.01 (0.01-0.01)	0.01 (0.01-0.01)	0.26
**C12OH**	0.01 (0.01-0.01)	0.01 (0.01-0.01)	0.77
**C14OH**	0.01 (0.00-0.01)	0.01 (0.01-0.01)	0.84
**C16OH**	0.01 (0.00-0.01)	0.01 (0.01-0.01)	0.43
**C18OH**	0.01 (0.00-0.01)	0.01 (0.00-0.01)	0.94
**C5:1**	0.01 (0.01-0.01)	0.01 (0.01-0.02)	0.22
**C6:1**	0.03 (0.03-0.04)	0.03 (0.03-0.04)	0.005
**C8:1**	0.01 (0.01-0.02)	0.01 (0.01-0.01)	1.00
**C10:1**	0.02 (0.01-0.02)	0.02 (0.01-0.03)	0.15
**C12:1**	0.01 (0.01-0.01)	0.01 (0.01-0.01)	0.61
**C14:1**	0.01 (0.00-0.01)	0.01 (0.00-0.01)	0.64
**C16:1**	0.01 (0.00-0.01)	0.01 (0.01-0.01)	0.39
**C16:1OH**	0.01 (0.00-0.01)	0.01 (0.00-0.01)	0.59
**C18:1**	0.01 (0.00-0.01)	0.01 (0.01-0.01)	0.74
**C18:1OH**	0.01 (0.01-0.01)	0.00 (0.00-0.00)	0.06
**C10:2**	0.01 (0.01-0.02)	0.02 (0.01-0.02)	0.05
**C14:2**	0.01 (0.01-0.01)	0.01 (0.01-0.01)	0.94
**C18:2**	0.01 (0.01-0.02)	0.01 (0.01-0.01)	0.76
**Total esteried carnitines**	4.0 (3.6-4.8)	5.8 (4.3-6.3)	0.004
**Total esterified/C0**	0.67 (0.58-0.76)	0.64 (0.59-0.70)	0.35

Ala: alanine; Val: valine; Xle: Leucine /Isoleucine; Met: methionine; Phe: phenylalanine; Tyr: tyrosine: Asp: aspartate; Glu: glutammate; Gly: glycine; Orn: ornitine; Cit: citrulline; Arg: arginine; C0 Free carnitine; C2 Acetylcarnitine; C3 Propionylcarnitine; C4 Butyrylcarnitine/isobutyrylcarnitine; C5 Isovalerylcarnitine + methylbutyrylcarnitine; C6 Hexanoylcarnitine; C8 Octanoylcarnitine; C10 Decanoylcarnitine; C12 Dodecanoylcarnitine; C14 Tetradecanoylcarnitine; C16 Hexadacanoylcarnitine; C18 Octadecanoylcarnitine; C3DC Malonylcarnitine; C4DC Methylmalonilcarnitine; C5DC Glutarylcarnitine; C6DC Methylglutarylcarnitine; C8DC Octanedioylcarnitine; C10DC Decanedioylcarnitine; C4OH 3-Hydroxybutyrylcarnitine; C5OH 3-Hydroxyisovalerylcarnitine/3-hydroxy-2-methylbutyrylcarnitine; C6OH 3-Hydroxyhexanoylcarnitine; C12OH 3-Hydroxydodecanoylcarnitine; C14OH 3-Hydroxytetradecanoylcarnitine; C16OH 3-Hydroxyhexadecanoylcarnitine; C5:1 Tiglylcarnitine; C6:1

Hexenoylcarnitine; C8:1 Octenoylcarnitine; C10:1 Decenoylcarnitine; C12:1 Dodecenoylcarnitine; C14:1 Tetradecenoylcarnitine; C16:1 Hexadecenoylcarnitine; C16:1OH 3-Hydroxyhexadecenoylcarnitine; C18:1 Octadecenoylcarnitine; C18:1OH 3-Hydroxyoctadecenoylcarnitine; C10:2 Decadienoylcarnitine; C14:2 Tetradecadienoylcarnitine; C18:2 Octadecadienoylcarnitine

Supplementary Figure 1. Dendrogram for hierarchical cluster analysis of metabolomic data

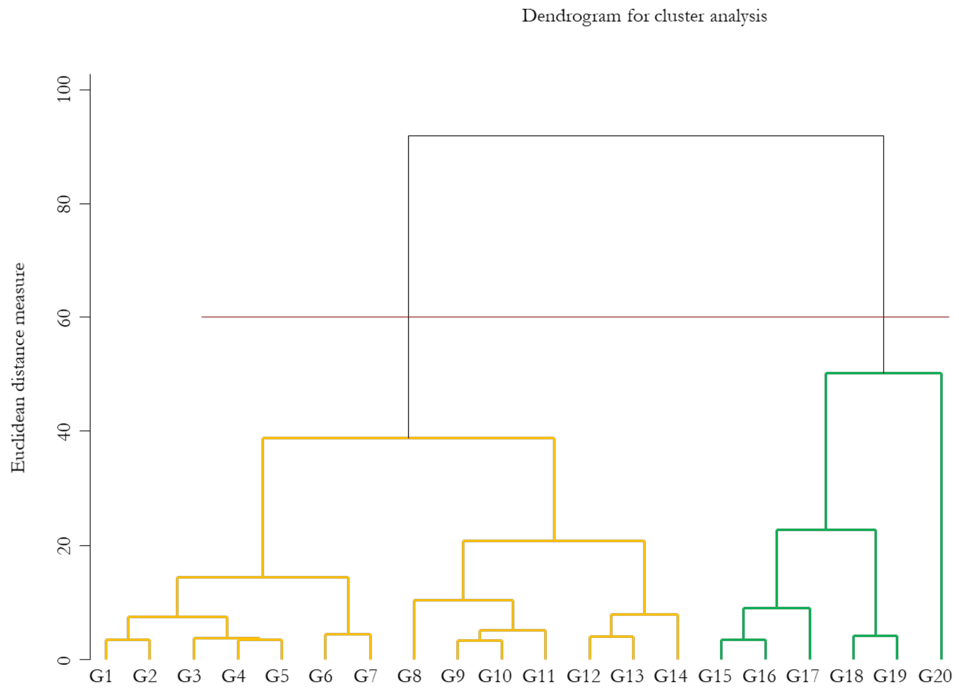

Supplement: Supplementary file 1 [file biomedicines-11-02830-s001.zip › biomedicines-2606846-supplementary.pdf]
